# Opportunistic Co-infections in Pulmonary Tuberculosis

**DOI:** 10.7759/cureus.98453

**Published:** 2025-12-04

**Authors:** Nuno Oliveira, Vania Junqueira, Rosa Amorim

**Affiliations:** 1 Internal Medicine, Unidade Local de Saúde do Oeste - Caldas da Rainha, Caldas da Rainha, PRT

**Keywords:** aduly pulmonology, bacterial coinfection, public health problems, streptococcus parasanguinis, streptococcus pseudoporcinus, tb – tuberculosis

## Abstract

Pulmonary tuberculosis is a pathology caused by *Mycobacterium tuberculosis* or Koch’s bacillus. Some patients with tuberculosis present co-infection with *Streptococcus* genus bacteria. Nevertheless, this co-infection is very rare, mainly in HIV patients. Here, we report the case of a 38-year-old male patient, previously healthy, who presented with subacute cough and fever, in whom two uncommon *Streptococcus* species were identified. Because symptoms persisted despite directed antibiotic therapy, bronchofibroscopy was performed, and *M. tuberculosis *was identified in the lavage. After diagnosis, the patient was referred to the Center for Pneumological Diseases for continued treatment.

## Introduction

Tuberculosis (TB) has remained a significant global health concern for decades. Before the severe acute respiratory syndrome coronavirus 2 (SARS-CoV-2) pandemic, it was the ninth leading cause of death worldwide [1-6]. Estimates suggest that one quarter of the global population is contaminated with active or latent TB, although this estimate requires caution because of marked regional variability and limited quantitative data, particularly in low-income regions where this pathology is highly prevalent [1,2]. 

The global incidence has increased to 134 cases per 100,000 inhabitants (2023 data), reversing the decline observed until 2020. India, Indonesia, China, the Philippines, Pakistan, Nigeria, Bangladesh, and the Democratic Republic of the Congo accounted for most cases. Approximately 83% of new diagnoses involve respiratory TB [1-3].

The classic triad of fever, night sweats, and weight loss occurs in approximately 75%, 45%, and 55% of patients, respectively, while a persistent non-remitting cough is reported in 95% [2]. Pulmonary TB symptoms can vary considerably, and some studies report that only one-third of patients have pulmonary respiratory pathology [2]. Cough may be productive or nonproductive. Sputum may appear mucoid, mucopurulent, blood-stained, or present as hemoptysis [1-4]. Radiographic findings range from a normal chest X-ray to upper-lobe opacities, diffuse opacities, consolidation, cavities, miliary pattern, thoracic lymphadenopathy, or pleural effusion. These represent only lung parenchymal manifestations. This paper does not address extrapulmonary TB [1-4]. 

Because TB presents with numerous symptoms and forms, diagnosis begins with thorough clinical history collection supported by suggestive imaging and sputum sampling [3]. Skin tests have fallen out of use because of poor precision in patients with prior disease contact or vaccination. The alternative Interferon-Gamma Release Assay (IGRA) requires approximately 24-48 hours, but in the authors' health unit, it takes about three weeks for results to be available [3]. Consequently, sputum analysis combined with imaging has become increasingly important. No diagnostic method is infallible, and false negatives occur. Pulmonary TB is frequently associated with secondary bacterial, viral, and fungal infections [6,7]. Patients with TB often have compromised immunity, which increases susceptibility to secondary infections due to complex host-pathogen interactions and structural lung changes [4-6]. 

Bir et al. (2024) identified bacterial coinfections in 19 of 174 sputum samples from confirmed patients with pulmonary TB. *Pseudomonas aeruginosa* was the most common (36.84%), followed by *Acinetobacter baumannii* (31.57%), *Klebsiella pneumoniae* (26.31%), and *Stenotrophomonas maltophilia* (5.28%). Resistance patterns resembled those seen in hospital-acquired infections [7]. Additional co-infectious agents may appear depending on environmental and host factors. *Streptococcus *genus co-infection is very rare and is mainly associated with HIV patients [6].

## Case presentation

A male patient aged 38 years, Brazilian, without recent travel and employed as a company administrator, was admitted with fever, productive and emetizing cough, and pleuritic pain evolving for about three weeks. These symptoms were accompanied by night sweats but not by weight loss or chest pain. The complementary study demonstrated increased inflammatory parameters and elevated erythrocyte sedimentation rate (ESR), as shown in Table 1. 

**Table 1:** Inflammatory parameters at patient's admission showing marked elevation

Inflammatory parameter	Measured value	Reference value
Leucocyte (cells/µL)	20,700	4,000-10,000
Neutrophil (cells/µL)	19,020	1,500-8,000
Procalcitonin (PCT) (ng/mL)	0.89	<0.50
C-reactive protein (CRP) (mg/dL)	31.9	<0.5
Erythrocyte sedimentation rate (ESR) in the first hour (mm/h)	120	12-14

**Figure 1:**
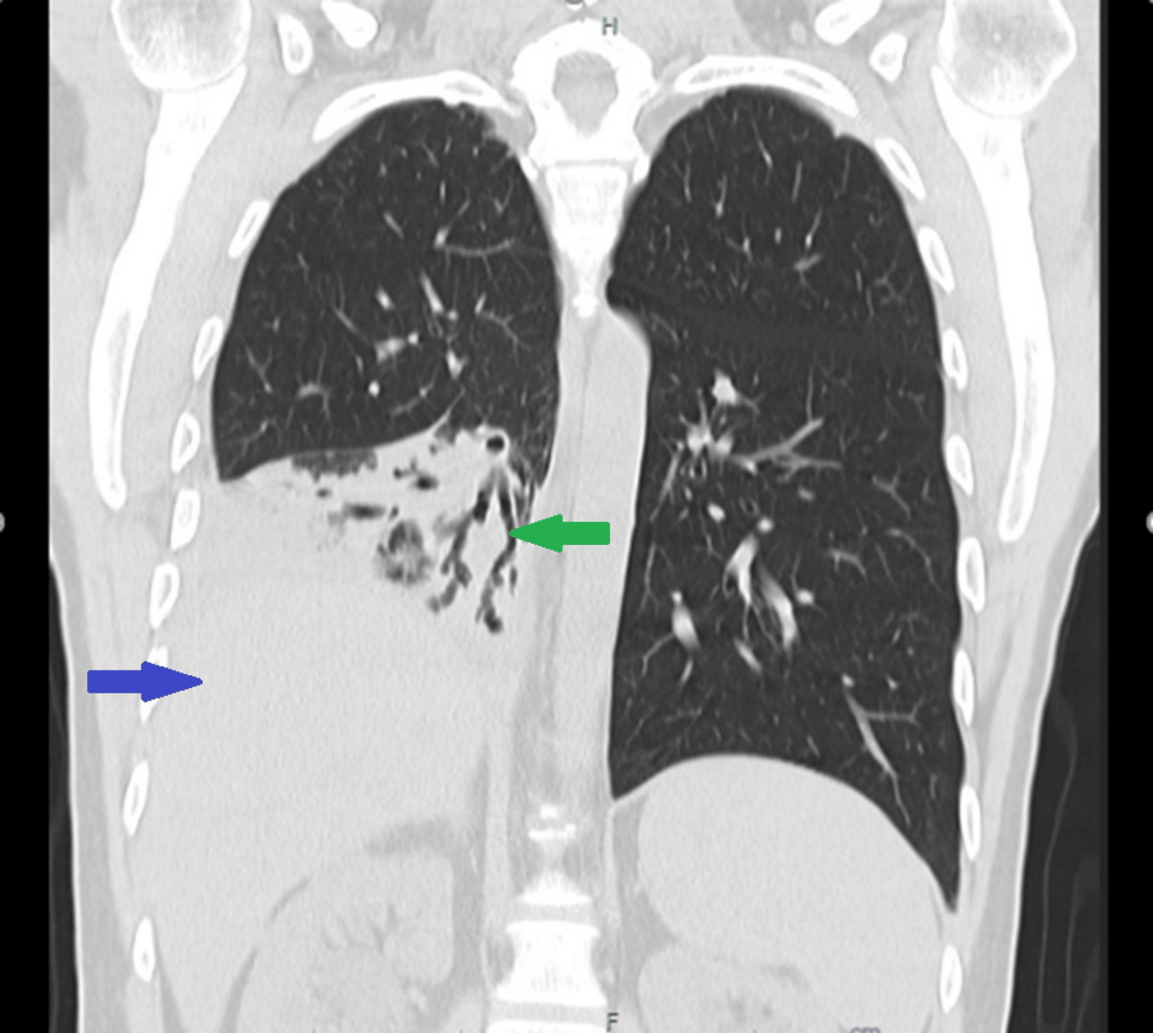
Lung CT scan showing right lobe condensation (green arrow) and pleural effusion (blue arrow)

**Figure 2:**
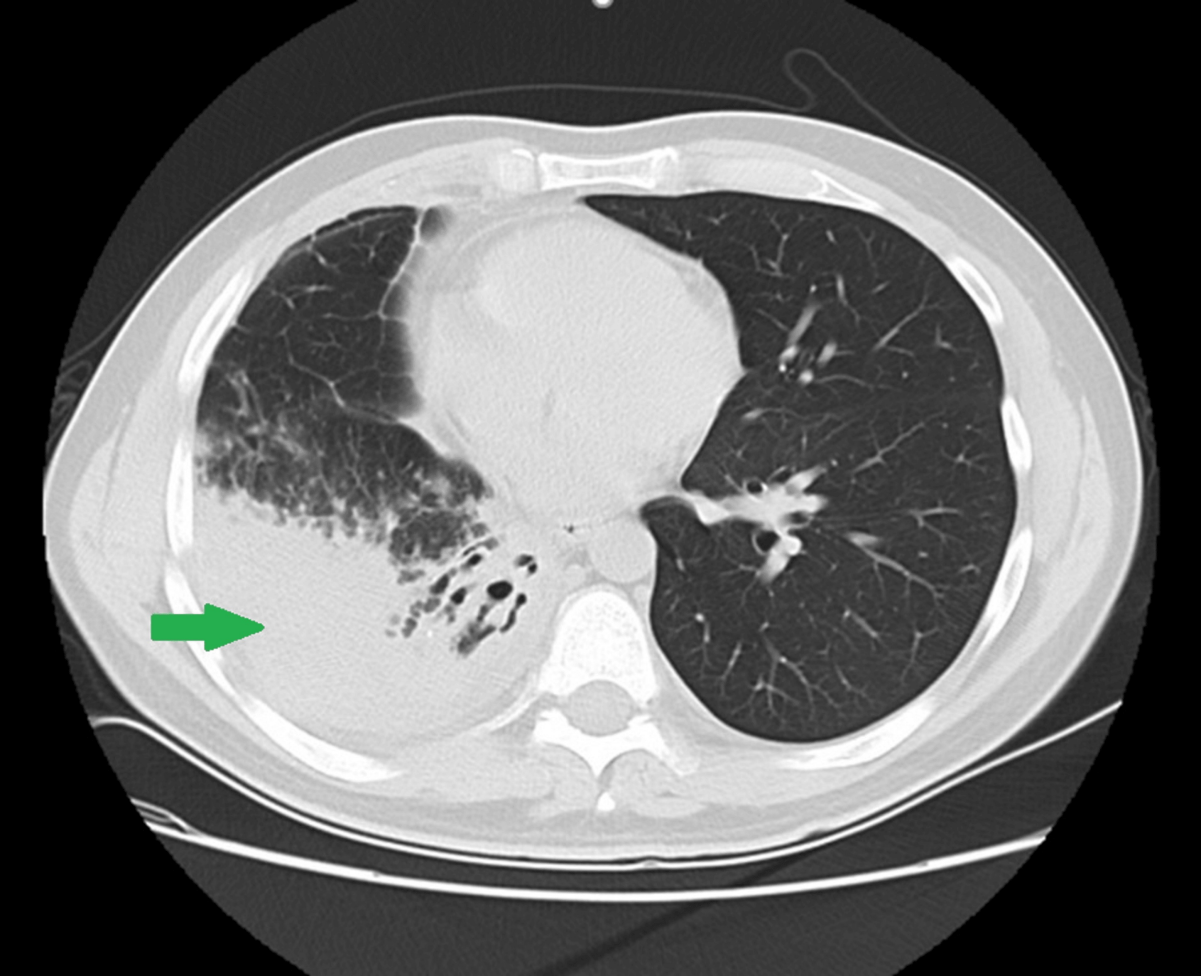
Lung CT scan showing right pleural effusion (green arrow)

Thoracic X-ray demonstrated pleural effusion and a condensation in the right lobe. A confirmatory chest CT scan showed marked reduced transparency with consolidation extending to the right lower lobe and an air bronchogram, findings that suggested an active inflammatory or infectious pneumopathy, reactive intracranial lymph nodes in the right hilum, and absence of pleural effusion. Four days later, a repeat chest CT scan reported pleural effusion with a cisural component in the right lobe that measured 27 mm in thickness in the costophrenic recess, as shown in Figures 1, 2.

Sputum culture isolated *Streptococcus pseudoporcinus*, with no available antibiotic sensitivity test. Piperacillin-tazobactam was initiated. Owing to a lack of improvement, a subsequent sputum culture isolated *Streptococcus parasanguinis*. A total of six sputum cultures were performed, but only two were not contaminated. The isolation results and their respective antibiotic sensitivity are presented in Table 2. 

**Table 2:** Sputum isolates and their respective antibiotic sensitivity

Sputum sample	Isolate	Antibiotic sensitivity test
First sample	Streptococcus pseudoporcinus	With no breakpoints for antibiogram
Fourth sample	Streptococcus parasanguinis	Sensitive to cefotaxime, clindamycin, teicoplanin, and vancomycin; resistant to ampicillin; intermediate resistance to benzylpenicillin

**Figure 3:**
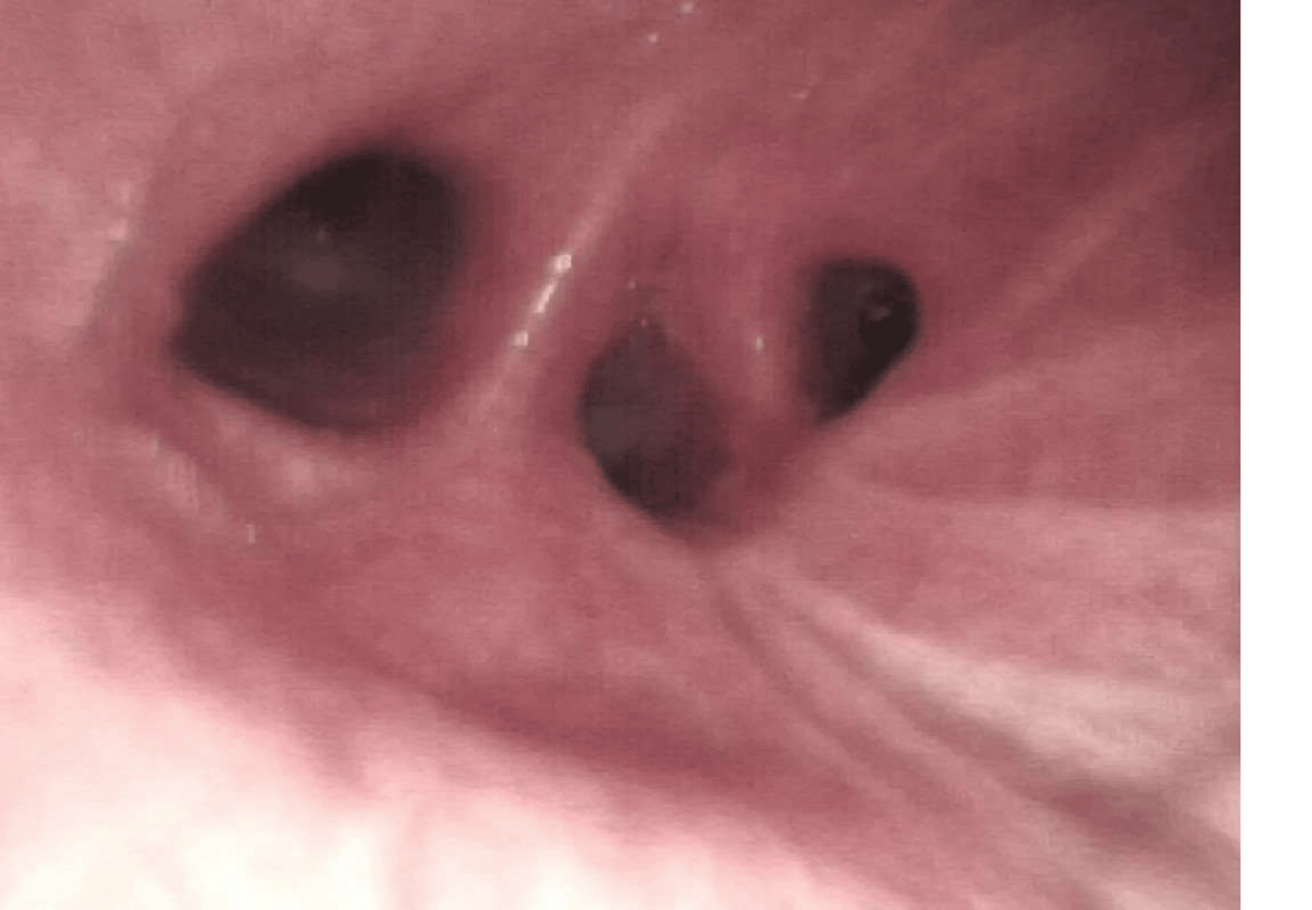
BFC image showing mucosal edema and hyperemia in the right inferior lobe and the right median lobe BFC, bronchofibroscopy.

To rule out endocarditis, an echocardiogram was performed, which showed no evidence of alterations. The patient was evaluated with Pulmonology and underwent bronchofibroscopy with lavage, and the procedure findings are presented in Figure 3. Aside from hyperemia and edema in the right lobes, no additional alteration was observed.

*Mycobacterium tuberculosis* was identified in bronchial secretions. There were no isolations in blood and urine culture, and there was no acid-fast bacillus (AFB) identification in the sputum (three sputum collections). IGRA was positive, and the result was available three weeks post-discharge. Anti-tuberculous drugs were started during hospitalization, which continued post-discharge. The patient was referred to a pneumological diagnostic unit.

## Discussion

The clinical presentation of hemoptysis for three weeks, night sweats, and an ESR of 120 mm/h suggested TB [2,3]. However, during the etiological investigation, the bacterial isolations of *S. pseudoporcinus* and *S. parasanguinis* introduced diagnostic uncertainty and contributed to the delay. While *S. parasanguinis* is a commensal organism of the mouth and may reflect contamination during sample collection, this explanation is less likely for *S. pseudoporcinus* [6-8]. Khan et al. (2020) described four cases of infection by this agent, including endocarditis and pneumonia with empyema, and some samples exhibited multi-antibiotic resistance [8]. Therefore, community-acquired pneumonia due to *S. pseudoporcinus* could not be ruled out. 

Bir et al. (2024) reported that bacterial co-infection in pulmonary TB occurs in approximately 10% of cases and is more common in male patients, according to [7]. Frequently associated organisms include *P. aeruginosa*, *A. baumannii*, and *Stenotrophomonas maltophilia* [8]. Among streptococcal species, *S. pneumoniae* is most often linked to co-infection, particularly in immunocompromised patients [9]. No published cases were identified that associate *S. pseudoporcinus* with TB.

Because our laboratory did not have antibiogram testing, antibiotic therapy was empirical and produced favorable results. At that point, both a laboratory hypothesis and a clinical one remained plausible. Pulmonology was consulted for possible bronchofibroscopy. AFB was identified only in the alveolar lavage.

Co-infections are mainly reported in association with immunodeficiency [9-13]. Nevertheless, latent TB before this episode could not be excluded. In such cases, pneumonia caused by *S. pseudoporcinus* could theoretically trigger activation of latent TB. Another possibility involves an intermediary state of TB with disease progression and concurrent co-infection. According to Drain et al. (2018), the interaction between the host and *M. tuberculosis* is continuous, and intermediary states may exist with absent symptoms yet signs of progression [14]. Therefore, it is plausible that the patient had an intermediary form of TB co-infected with *S. pseudoporcinus*, and the symptoms were primarily attributable to the co-infecting agent. 

## Conclusions

Co-infection in a patient with TB represents a complex clinical challenge. On one hand, it adds additional symptoms and alters imaging and laboratory findings; on the other hand, it delays the initiation of anti-TB therapy while requiring additional medications. The main lesson is the importance of maintaining clinical reasoning. Even when cultural results identify a plausible cause, there are situations in which further investigation is warranted. In this patient, despite the isolation of two different streptococci, the prolonged clinical course and ESR of 120 mm/h supported the suspicion of another underlying pathology. TB was diagnosed only through flexible bronchoscopy.
